# Sustainable Lime–Silica Materials Based on Waste Packaging Glass

**DOI:** 10.3390/ma19183970

**Published:** 2026-09-18

**Authors:** Katarzyna Borek

**Affiliations:** Faculty of Civil Engineering and Architecture, Kielce University of Technology, Al. Tysiąclecia Państwa Polskiego 7, 25-314 Kielce, Poland; k.komisarczyk@tu.kielce.pl

**Keywords:** autoclaved materials, waste packaging glass, glass color, sustainable construction materials, quartz sand replacement, Spearman’s rank correlation

## Abstract

The use of waste packaging glass as a substitute for quartz sand may reduce the consumption of natural raw materials and provide an additional route for glass-waste valorization. However, the combined effects of glass color and replacement level on the relationships among the physical and mechanical properties of autoclaved lime–silica products remain insufficiently understood. This study therefore evaluated products in which 33%, 66%, and 100% of the quartz sand by mass was replaced with colorless, green, or brown waste packaging glass. Cylindrical specimens were autoclaved at 180 °C and a saturated steam pressure of 1.002 MPa for 8 h. Compressive strength, volumetric density, water absorption, and open porosity were determined, and their relationships were evaluated using Spearman’s rank correlation analysis and linear regression. Both glass color and replacement level affected the investigated properties, while complete replacement of quartz sand produced the most pronounced changes. The series containing 100% colorless waste glass exhibited the highest compressive strength, water absorption, and open porosity, together with the lowest volumetric density. The colorless glass series also produced the strongest linear fits, with R^2^ values of 0.9703 for compressive strength and volumetric density, 0.9892 for compressive strength and open porosity, 0.975 for water absorption and volumetric density, and 0.9473 for open porosity and volumetric density. The corresponding relationships for brown and green glass were generally weaker, with R^2^ values ranging from 0.6615 to 0.9131. These results indicate that glass color and replacement level can be treated as material design variables and that the identified relationships may support preliminary mixture screening within the investigated compositional range. Following independent validation, these relationships could also support the preliminary assessment of other soda–lime–silica glasses, such as flat glass, tempered automotive glass, or photovoltaic panel glass, while accounting for differences in particle-size distribution, material purity, and the presence of coatings. Waste packaging glass can therefore be considered as a partial or complete substitute for quartz sand, particularly in lightweight, strength-oriented products intended for dry or protected environments. However, the increased water absorption of the colorless glass series and the limited scope of the laboratory tests require further validation before structural applications or applications involving moisture exposure can be recommended.

## 1. Introduction

Glass packaging is one of the most developed material-recycling streams in Europe. According to the European Container Glass Federation, the average EU collection rate for glass packaging reached 80.1% in 2021, while the Close the Glass Loop partnership aims to increase this rate to 90% by 2030. Nearly 12 million metric tons were collected, and 91% of effectively recycled glass packaging was returned to bottle and jar production [1,2]. Closed-loop remelting remains the preferred route; however, complementary outlets are needed for glass fractions that cannot be efficiently returned to container production. In Poland, the National Waste Management Plan 2028 identifies insufficient processing capacity, particularly for waste glass, and inadequate integration between packaging and municipal waste-management systems [3]. The use of waste packaging glass in construction materials may therefore provide an additional valorization route while reducing demand for natural mineral resources.

In construction materials, waste glass can be used as an aggregate or inert filler, as a finely ground reactive addition, or as a silica-bearing precursor in alkali-activated materials (AAMs) and geopolymers. Its function depends on particle-size distribution, chemical composition, glass purity, activator chemistry, and curing conditions. Reviews of cementitious and alkali-activated systems show that waste glass can contribute as a precursor, filler, or source of soluble silica [4,5]. Glass color is not necessarily a dominant factor at moderate contents because container glasses commonly have a similar soda-lime–silica base composition; nevertheless, a recent study of high-volume glass-powder cement pastes specifically examined color as a potential source of performance differences [6]. These findings justify considering color as a material variable, but their mechanisms cannot be transferred directly between Portland-cement, alkali-activated, and autoclaved lime–silica systems.

The use of comparatively coarse waste-glass aggregate in Portland-cement concrete also requires consideration of the alkali–silica reaction (ASR), particularly in moist environments. Gholampour et al. demonstrated that ASR expansion in concrete containing crushed-glass sand depends on the surrounding binder system and can be mitigated by an appropriate combination of fly ash and ground granulated blast-furnace slag [7]. Calcium nitrate has likewise been shown to reduce ASR in Portland-cement mortars by forming a passivating layer on reactive aggregate surfaces [8]. However, the present lime–silica products contain no Portland cement and are cured hydrothermally; consequently, the effectiveness of these mitigation strategies cannot be assumed and would require separate experimental verification.

Autoclaved lime–silica products are formed from quicklime, quartz sand, and water. During hydrothermal treatment, Ca(OH)_2_ reacts with dissolved silica to form calcium silicate hydrate (C-S-H) phases, including crystalline tobermorite. Their performance depends on the Ca/Si ratio, silica dissolution kinetics, particle packing, compaction, autoclaving conditions, and the resulting pore structure [9,10]. Replacing quartz sand with waste packaging glass changes several of these factors simultaneously because glass supplies amorphous silica and alkalis and differs from quartz in surface characteristics and dissolution behavior.

Earlier studies confirmed the technological feasibility of using waste packaging glass as a quartz-sand substitute in autoclaved lime–silica products. Borek et al. reported that increasing replacement with glass cullet reduced volumetric density and increased compressive strength, with the largest changes occurring at complete replacement [11]. A subsequent matched comparison of colorless, brown, and green packaging glass showed that both glass color and replacement level affected mechanical and physical properties [12]. Microstructural observations further indicated that waste glass modifies the formation and morphology of calcium silicate hydrates, while Szudek et al. found that waste glass powder promoted amorphous and semicrystalline C-S-H and hindered the formation of well-crystallized tobermorite [13].

Recent independent studies have strengthened the phase-based interpretation of these effects. Gao et al. showed that waste glass can increase the strength of quartz-sand autoclaved materials, but that the response depends on the Ca/Si ratio and replacement level: lower glass contents may promote tobermorite, whereas high contents favor amorphous, highly polymerized C-S-H [14]. Their comparison of waste glass and fly ash as silica sources further confirmed that precursor reactivity governs hydration-product development and performance [15]. In autoclaved aerated concrete, Straub et al. observed improved strength-to-density performance at moderate waste-glass-powder contents, whereas higher contents reduced tobermorite crystallinity and increased drying shrinkage [16]. Collectively, these studies show that complete replacement cannot automatically be treated as a proportional continuation of the behavior observed at lower glass contents.

Despite this progress, previous studies have primarily quantified changes in individual properties or compared series mean values. A matched, individual-measurement assessment integrating compressive strength, volumetric density, water absorption, and open porosity for colorless, brown, and green waste packaging glass at both partial and complete quartz-sand replacement remains limited. This gap is important because increased compressive strength may coexist with reduced volumetric density, increased open porosity, and increased water absorption, suggesting that the load-bearing solid skeleton and the connected pore network develop differently. Earlier investigations [11,12,17] established the effects of glass color and content but did not determine whether these four properties exhibit consistent, color-dependent associations or whether 100% replacement constitutes a distinct response regime.

Correlation-based methods have previously been used to investigate relationships among material properties in concrete and geopolymer systems [18,19]. In the present study, Spearman’s rank correlation and linear regression are used as supporting analytical tools rather than as the sole source of novelty. Spearman’s coefficient quantifies monotonic associations without assuming normality or linearity, whereas regression describes only the linear component of selected relationships within the investigated compositional range.

Accordingly, the present study evaluates a matched experimental matrix comprising colorless, brown, and green waste packaging glass at 33%, 66%, and 100% quartz-sand replacement under identical mixing, compaction, and autoclaving conditions. Its principal contribution is a four-property response framework based on individual measurements that compares the coordinated evolution of compressive strength, volumetric density, water absorption, and open porosity; identifies color-dependent mechanical-moisture trade-offs; and tests whether complete replacement represents a distinct material response. The resulting relationships may support preliminary selection of a glass stream and replacement level, but they are not universal predictive models. Because only one source of each glass color, one particle-size distribution, and laboratory-scale specimens were investigated, independent validation is required. Similar overall proportions of SiO_2_, Na_2_O, and CaO alone do not guarantee equivalent behavior, because coatings, polymer interlayers, coloring oxides, impurities, particle characteristics, and material purity may affect hydrothermal reactions and pore development.

## 2. Materials and Methods

### 2.1. Materials

As part of this study, samples of lime–silica products were prepared. Their basic composition consisted of quartz sand, lime, and water. Crushed glass from glass packaging was used as an aggregate substitute and added to the tested products in three color variants: colorless, green, and brown. Data characterizing the individual components of the lime–silica mixture are presented in Table 1.

The chemical composition of aggregates (sand and waste glass) was tested in accordance with ISO 29581-2:2010 [21] and PN-EN 196-2:2013-11, p. 5 [22], which was the applicable edition when the tests were performed. The results are presented in Table 2.

### 2.2. Testing Methods

Ten series were prepared: one reference series without waste glass and nine modified series containing colorless, green, or brown waste packaging glass at three quartz-sand replacement levels: 33%, 66%, and 100% by mass. Twelve cylindrical specimens, each measuring 25 mm in diameter and height, were produced for each series, giving a total of 120 specimens. Six specimens from each series were subjected to compressive strength testing. The remaining six specimens were used to determine bulk density, water absorption, and open porosity. The applied testing procedures are summarized in Table 3.

The normality of the data distributions was assessed using the Shapiro–Wilk test. Most datasets did not meet the assumption of normality (*p* < 0.05); the only exception was volumetric density for the green glass series. Spearman’s rank correlation coefficient (r_s_) was therefore selected instead of Pearson’s coefficient because it does not require normally distributed data and measures monotonic relationships without assuming linearity. Spearman’s method was applied consistently to all glass series to ensure comparability.

The correlation analyses were based on individual measurements rather than series mean values. For each dataset corresponding to a given glass color, 24 individual observation pairs were included: six for the reference composition and six for each quartz sand replacement level of 33%, 66%, and 100%. Two-sided *p*-values were calculated, with *p* < 0.05 indicating statistical significance. Within-series variability was described using standard deviations.

Spearman’s correlation coefficient ($r_{s}$) was calculated using Equation (4) [25]:

(4) $$r_{s}=1-\frac{6\times \sum d_{i}^{2}}{n(n^{2}-1)}$$

where

n is the number of observations;

d_i_ is the difference between the ranks assigned to the ith pair of observations, calculated as d_i_ = RX_i_ − RY_i_;

RX_i_ − RY_i_ are the ranks assigned to the corresponding values of properties X and Y. The coefficient ranges from −1 to +1, with values close to +1 or −1 indicating a strong positive or negative monotonic relationship, respectively, and values close to 0 indicating a weak or absent monotonic relationship.

Linear regression was conducted separately to characterize the linear component of the relationships. The coefficient of determination (R^2^) was interpreted exclusively as a measure of linear model fit and not as evidence of causality or validated predictive capability.

### 2.3. Preparation of Samples

**Figure 1:**
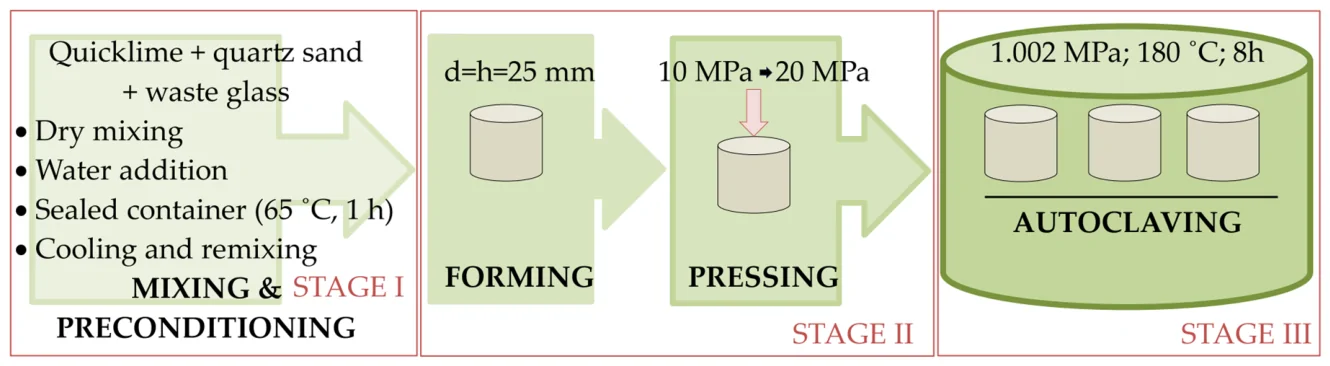
Schematic representation of the three-stage sample preparation procedure: (**I**) mixing and preconditioning, (**II**) forming and two-stage pressing, and (**III**) autoclaving.

The sample preparation procedure comprised three stages, as illustrated in Figure 1. In Stage I, the dry components were weighed and manually mixed with a metal spoon for 3 min using a continuous, uniform clockwise motion. These components comprised quicklime and an aggregate consisting of quartz sand, waste packaging glass, or a mixture of both, depending on the sample series. The waste packaging glass was used in three color variants: colorless, green, and brown. Water was then added to obtain a moisture content of 6–8%. The resulting mass was transferred to a tightly sealed 1 L glass container and maintained at 65 °C for 1 h. It was subsequently cooled to room temperature (21 °C) and mixed again to ensure uniformity. In Stage II, cylindrical samples with a diameter and height of 25 mm were formed. The prepared mixture was placed in a steel mold and compacted using a hydraulic press in two stages: first at 10 MPa and, after venting, at 20 MPa. Each pressure level was maintained for 1 min. Stage III consisted of autoclaving. The samples were heated to 180 °C until a saturated steam pressure of 1.002 MPa was reached. The main autoclaving stage was conducted at 180 °C and 1.002 MPa for 8 h. The time course of the autoclaving process is presented in [17]. The hydrothermal treatment was conducted in a laboratory-scale high-pressure autoclave (Parr Instrument Company, Moline, IL, USA) equipped with a Parr 4848M reactor controller (Parr Instrument Company, Moline, IL, USA). Finally, the autoclave and specimens were allowed to cool for 12 h to an ambient temperature of approximately 21 °C, after which the specimens were removed.

**Figure 2:**
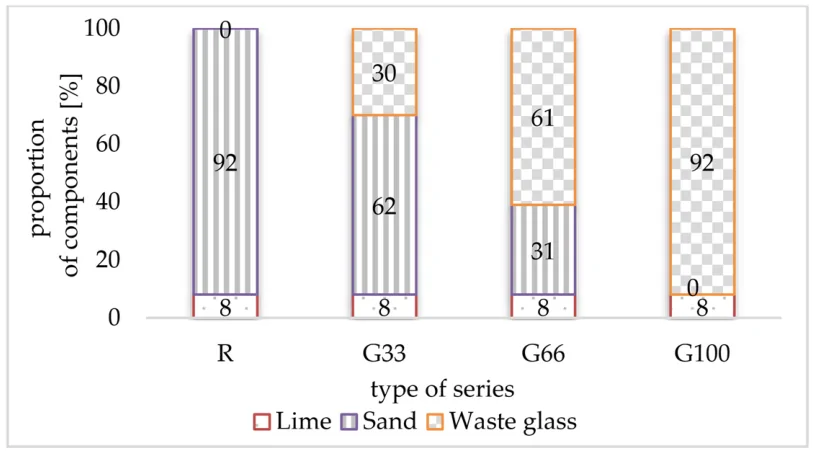
Mix compositions of the reference and waste-glass-modified series, expressed as percentages by mass of the dry components.

Figure 2 presents the proportions of quartz sand, lime, and waste packaging glass used in the experimental mixtures. The reference samples, featuring the classic composition without waste glass, were designated as R. The modified series were identified according to the color of the waste packaging glass: WG for colorless glass, GG for green glass, and BG for brown glass. The numerical suffix indicates the percentage of quartz sand replaced by waste glass by mass. Accordingly, 33 and 66 denote replacement levels of 33% and 66%, respectively, whereas 100 indicates the complete replacement of quartz sand.

## 3. Results and Discussion

**Figure 3:**
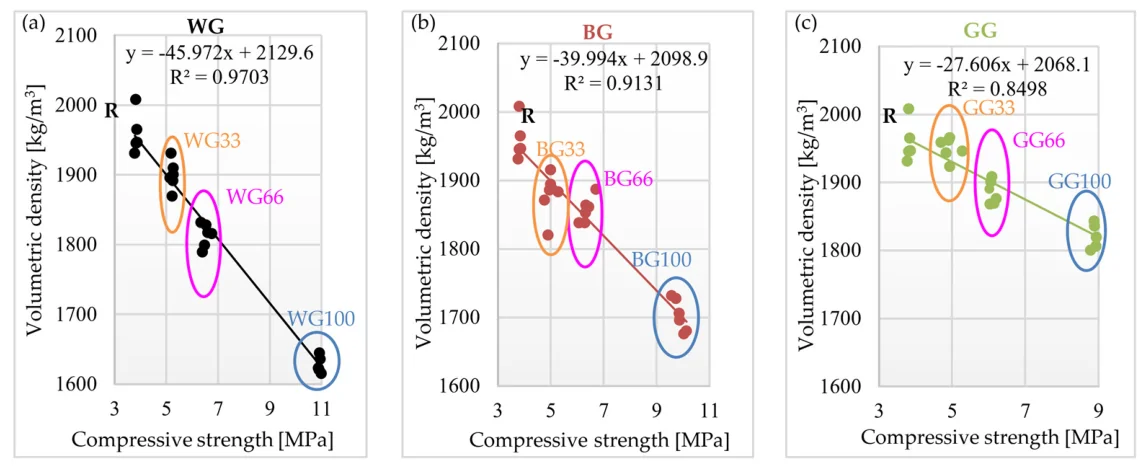
Relationships between compressive strength and volumetric density for samples containing: (**a**) colorless glass (WG); (**b**) brown glass (BG); and (**c**) green glass (GG).

**Figure 4:**
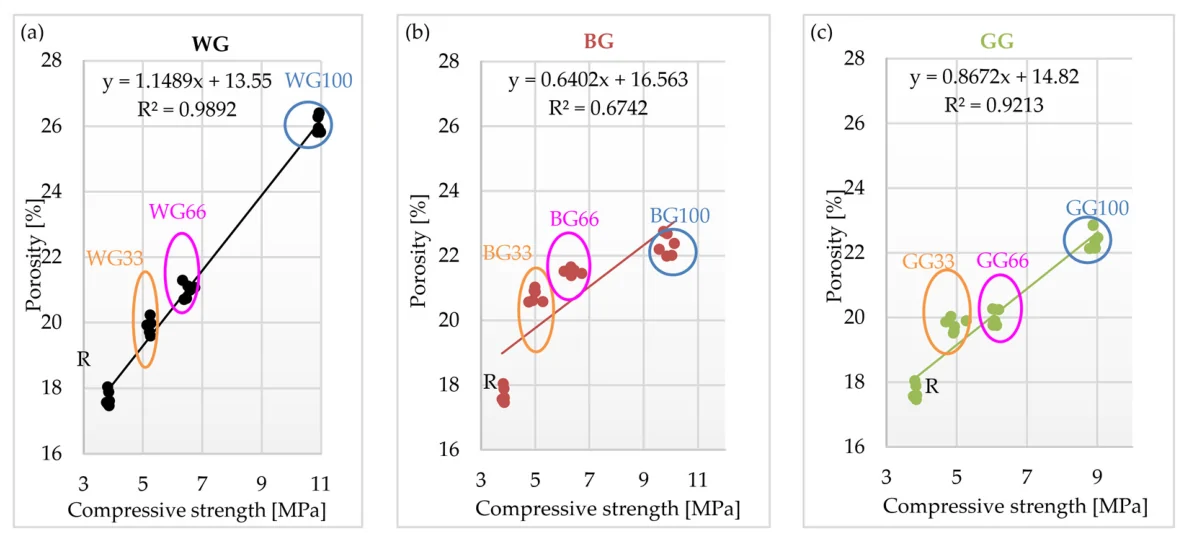
Relationships between compressive strength and open porosity for samples containing: (**a**) colorless glass (WG); (**b**) brown glass (BG); and (**c**) green glass (GG).

**Figure 5:**
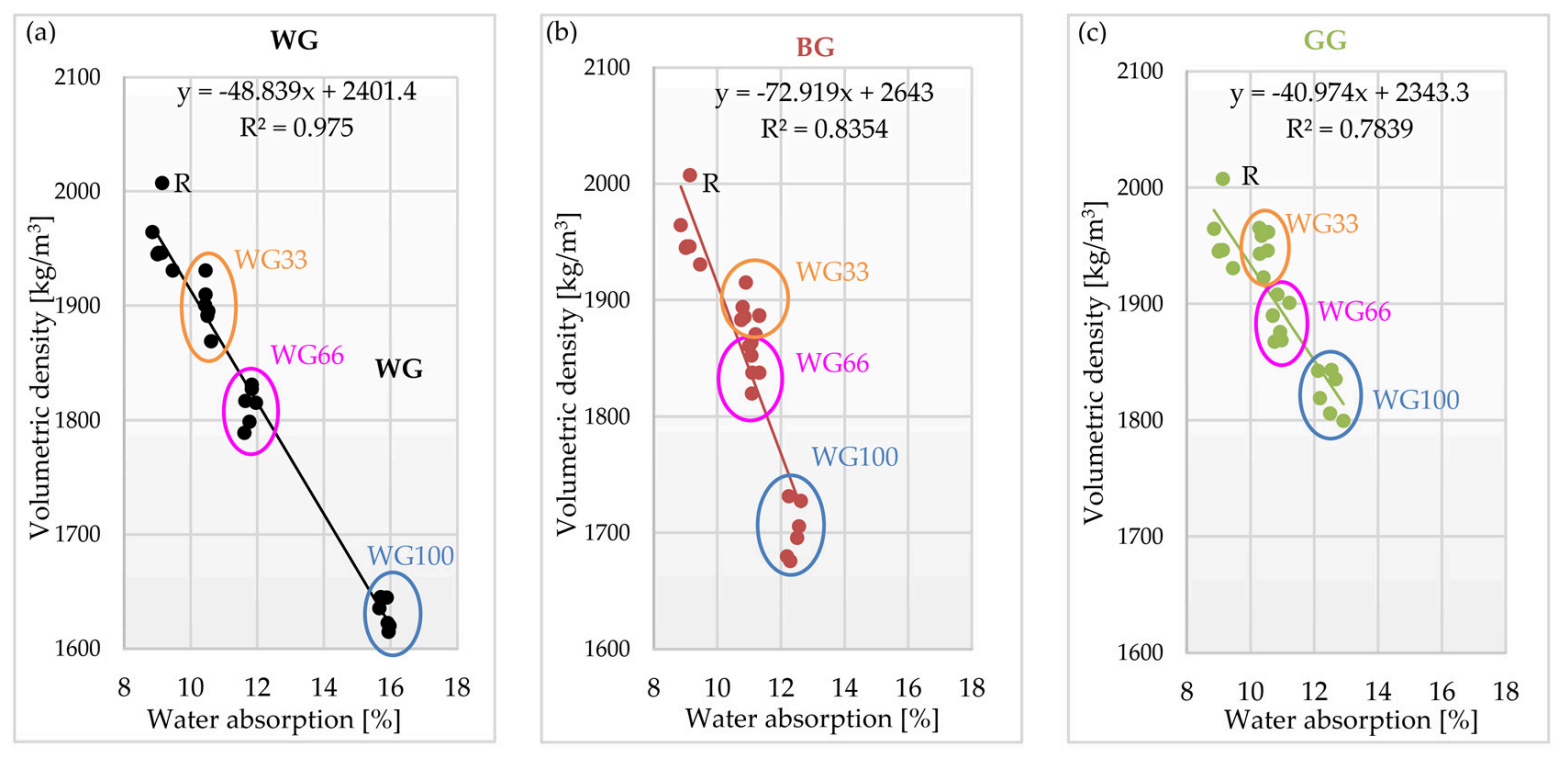
Relationship between volumetric density and water absorption for samples containing: (**a**) colorless glass (WG); (**b**) brown glass (BG); and (**c**) green glass (GG).

**Figure 6:**
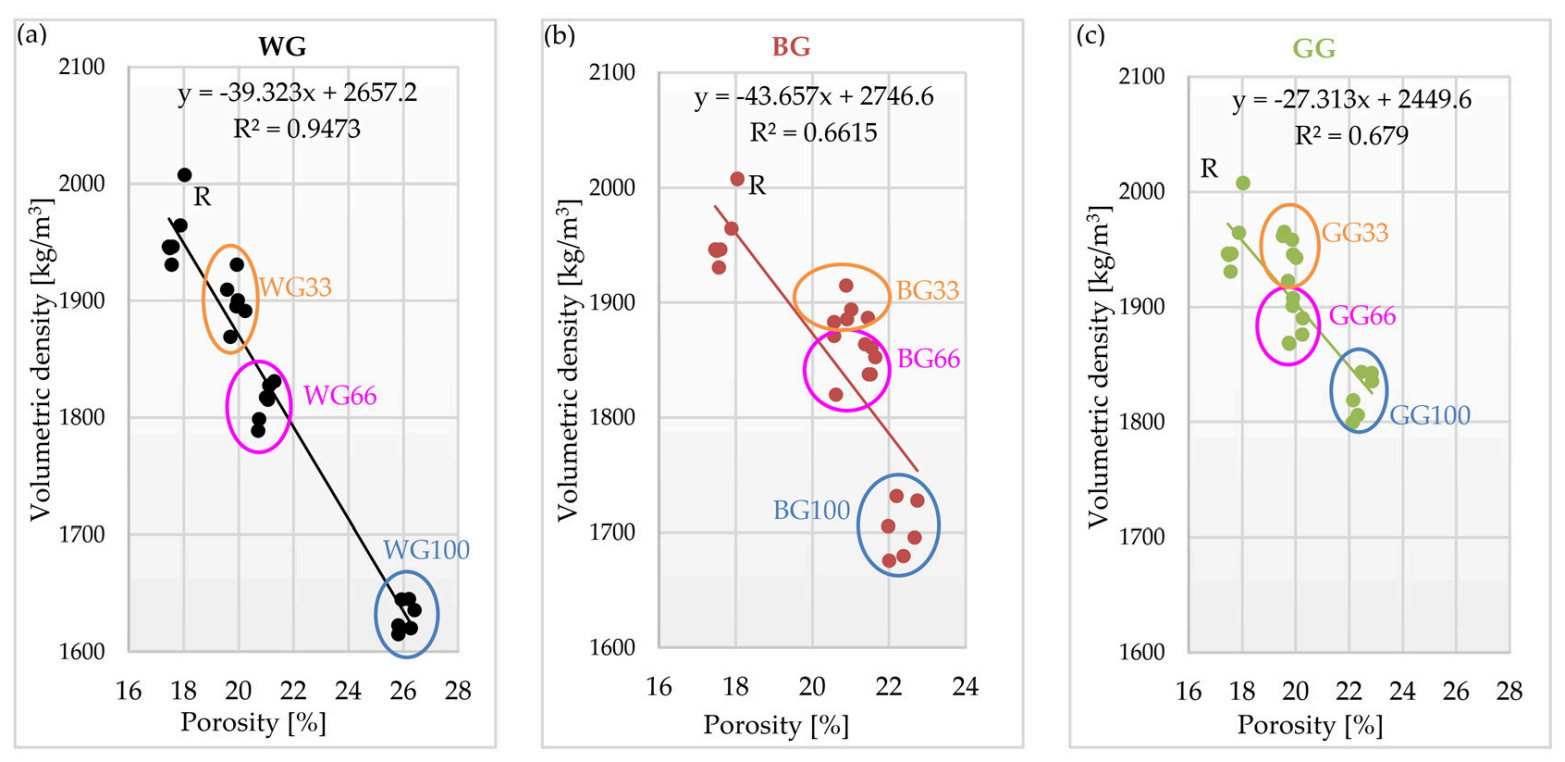
Relationship between volumetric density and open porosity for samples containing: (**a**) colorless glass (WG); (**b**) brown glass (BG); and (**c**) green glass (GG).

Figure 3, Figure 4, Figure 5 and Figure 6 present individual measurement values rather than series mean values. Each glass-specific analysis therefore included 24 observations.

The strength and direction of the relationships among the investigated properties of lime–silica products containing three types of waste glass were evaluated using Spearman’s rank correlation coefficient. The calculated r_s_ and *p*-values are presented in Table 4. Significant negative monotonic relationships were identified between compressive strength and volumetric density, with the strongest associations observed for the colorless glass (r_s_ = −0.925, *p* < 0.001) and brown glass series (r_s_ = −0.903, *p* < 0.001). The absolute values of the coefficients indicate that these properties were more strongly associated in the colorless and brown glass series than in the green glass series.

A significant positive relationship was observed between compressive strength and water absorption, with the strongest association found for the green glass series (r_s_ = 0.943, *p* < 0.001). Significant positive relationships were also identified between compressive strength and open porosity and between water absorption and open porosity. Overall, the strongest associations were generally observed for products containing colorless waste glass. In this series, volumetric density was strongly negatively associated with both water absorption (r_s_ = −0.938, *p* < 0.001) and open porosity (r_s_ = −0.905, *p* < 0.001). The strongest relationship among all investigated property pairs was observed between water absorption and open porosity for the colorless glass series (r_s_ = 0.950, *p* < 0.001). This result is consistent with the physical relationship between water uptake and the volume of interconnected pores. These correlations describe monotonic associations within the investigated dataset and should not be interpreted as evidence of causality or as universally validated predictive relationships.

Based on the diagram in Figure 3a–c, a strong negative relationship can be observed between compressive strength and volumetric density in all analyzed cases. The higher compressive strength was associated with lower volumetric density in the lime–silica products containing waste container glass. The coefficient of determination R^2^ was 0.9703 for the samples containing colorless glass and slightly lower for the samples containing brown and green glass, at 0.9131 and 0.8498, respectively. When compressive strength was used as the explanatory variable, these values indicate that the fitted linear models accounted for approximately 97%, 91%, and 85% of the variance in volumetric density for the colorless, brown, and green glass series, respectively. The results also indicate that the scatter of the reference and modified samples follows an approximately linear trend. A similar scatter pattern was observed for the lime–silica samples containing 100% glass replacement (WG100, BG100, and GG100). The distances between the data clusters representing the reference and 33% replacement series, and those representing the 33% and 66% replacement series, were comparable across the three glass colors. In contrast, a distinct separation was observed between the series containing up to 66% glass and those containing 100% glass, suggesting a pronounced change in volumetric density and compressive strength at complete aggregate replacement.

Figure 4a–c shows a strong positive relationship between compressive strength and open porosity in all analyzed cases. Higher compressive strength was associated with greater open porosity in the lime–silica products containing waste packaging glass. The coefficient of determination for the samples modified with colorless waste glass (a) was 0.9892, indicating the strongest fit of the linear model among the three glass types. For the samples containing brown glass, the measurement sets deviated from a straight-line trend, whereas for the samples containing green glass, the GG66 and GG100 sets were located close to the regression line. The intervals between the data sets corresponding to the 33% and 66% glass replacement levels were similar, regardless of glass color. A distinct separation was observed between the data for the 66% and 100% replacement levels, with the largest difference occurring between the WG66 and WG100 series. This pattern indicates that the relationship between open porosity and compressive strength was not equally linear for all glass colors, despite the overall positive association.

Figure 5a–c shows a strong negative relationship between water absorption and volumetric density in all analyzed cases. Higher volumetric density was associated with lower water absorption in the lime–silica products containing waste container glass. The coefficient of determination for the colorless glass series was the highest, at 0.975. Accordingly, when water absorption was modeled as a function of volumetric density, the fitted linear model accounted for approximately 98% of the variance in the results for the colorless glass series. For the brown and green glass series, the corresponding values were approximately 84% and 78%, respectively. The results indicate that water absorption and volumetric density were approximately linearly related for the WG and BG series, whereas the relationship was weaker and less clearly linear for the GG series. The spacing between the data points differed among the WG, BG, and GG samples. In the WG series, the distances between the reference and WG33 samples and between the WG33 and WG66 samples were similar, whereas the distance between the WG66 and WG100 samples was considerably greater.

Figure 6a–c shows a strong negative relationship between volumetric density and open porosity. Higher volumetric density was associated with lower open porosity in the lime–silica products containing waste container glass. The coefficients of determination for the WG series indicated the strongest relationship between open porosity and volumetric density. The R^2^ value was 0.9473 for the WG series, compared with 0.6615 for the BG series and 0.6790 for the GG series. Thus, the fitted linear model accounted for approximately 95% of the variance in open porosity for the colorless glass series, compared with approximately 66% and 68% for the brown and green glass series, respectively. The spacing between the result series for the WG, BG, and GG samples followed a pattern similar to that observed for the water absorption and volumetric density relationship. This similarity is consistent with the close relationship between water absorption and open porosity and suggests that these two parameters reflect related aspects of the pore structure of the investigated products.

The variability of the measurements is summarized in Table 5. The standard deviations ranged from 0.034 to 0.210 MPa for compressive strength, from 13.022 to 31.992 kg/m^3^ for volumetric density, from 0.07% to 0.30% for water absorption, and from 0.090% to 0.326% for open porosity. The relatively small within-series variability indicates good repeatability of the experimental results.

EN 771-2:2011+A1:2015 [26] does not specify a universal maximum water absorption value for calcium silicate masonry units. Instead, water absorption determined according to EN 772-21 is declared by the manufacturer when relevant to the intended application. The standard also distinguishes between protected and unprotected masonry and requires freeze–thaw performance to be considered when units may freeze under wet conditions. Therefore, the higher water absorption observed for the colorless glass series should not be interpreted as direct evidence of non-compliance. Rather, it indicates that these compositions may be more suitable for masonry protected from moisture and that their use under unprotected conditions requires verification using full-size masonry units.

The simultaneous increase in compressive strength and open porosity, particularly in the colorless glass series, indicates that the mechanical performance was not governed by the volume of water-accessible pores alone. Instead, compressive strength may also have depended on the development of the load-bearing solid skeleton and the quality of bonding at particle-contact zones. Previous XRD and SEM/EDS analyses performed on the same glass-containing specimens identified residual portlandite, calcite attributed to partial carbonation, tobermorite, and calcium silicate hydrates with different degrees of crystallinity [12]. The SEM observations also revealed voids between plate-like and irregular hydration products, particularly in the colorless-glass specimens. These findings provide a material-specific basis for interpreting the lower volumetric density and higher water absorption as consequences of a larger or more connected open-pore network.

Szudek et al. [13] similarly reported that sodium ions released from waste glass promoted the formation of amorphous and semicrystalline C–S–H phases while hindering their transformation into well-crystallized tobermorite. However, the finely ground glass powder used in their study also densified the matrix, demonstrating that the resulting microstructure depends not only on glass chemistry and alkali release but also on particle size, packing, and glass dissolution behavior. In the present specimens, glass replacement may therefore have modified the hydrate assemblage and strengthened bonding within the load-bearing skeleton while simultaneously altering particle packing and pore connectivity. Such a response could account for the coexistence of increased compressive strength, reduced volumetric density, and increased open porosity and water absorption.

The positive association between compressive strength and open porosity should therefore not be interpreted as evidence that increased porosity directly improves strength or as proof of a causal mechanism. Rather, both properties may have responded differently to changes induced by glass replacement. Because no new XRD, SEM/EDS, glass-dissolution, or pore-size-distribution analyses were performed in the present study, this explanation remains an evidence-based interpretation supported by previously published observations and requires further experimental verification.

## 4. Conclusions

Based on the findings of the study, the following conclusions can be drawn:

- Both glass color and quartz sand replacement level affected the coupled mechanical and pore-related response of the autoclaved lime–silica products. The 100% replacement level produced a marked departure from the trends observed at 33% and 66%, indicating that complete replacement was not merely a proportional continuation of the effects obtained at lower glass contents.
- Complete replacement with colorless glass produced the highest compressive strength and the lowest volumetric density, but also the highest water absorption and open porosity. Colorless glass therefore provided the most favorable strength-to-density response, accompanied by a clear moisture-related performance trade-off.
- All relationships included in the Spearman analysis were statistically significant (*p* < 0.001; n = 24 individual observation pairs per glass color). Compressive strength was negatively correlated with volumetric density, with r_s_ values of −0.925, −0.903, and −0.870 for colorless, brown, and green glass, respectively. It was positively correlated with open porosity, with corresponding r_s_ values of 0.937, 0.919, and 0.897. The strongest relationship was found between water absorption and open porosity for the colorless glass series (r_s_ = 0.950).
- The colorless glass series also exhibited the strongest linear model fits: *R*^2^ = 0.9703 for compressive strength–volumetric density, 0.9892 for compressive strength–open porosity, 0.9750 for water absorption–volumetric density, and 0.9473 for open porosity–volumetric density. These values describe linear model fit only and should not be interpreted as evidence of causality or as validation of a universal predictive model.
- The simultaneous increase in compressive strength and open porosity indicates that the load-bearing solid skeleton and the connected pore network developed differently. Based on previously published XRD and SEM/EDS observations for the same glass-containing specimens, C–S–H and tobermorite formed at particle contacts may have strengthened the solid skeleton, whereas glass-related changes in hydrothermal reactions, particle packing, and pore connectivity increased open porosity and water absorption. Because no new phase or microstructural analyses were performed, this explanation remains an evidence-based interpretation rather than direct proof of the mechanism.
- Colorless glass appears most suitable for lightweight, strength-oriented autoclaved products intended for dry environments or locations protected from moisture. Its higher water absorption does not directly indicate non-compliance because EN 771-2 does not specify a universal maximum value for this property. However, application under unprotected moisture exposure or freeze–thaw conditions requires additional testing on full-size masonry units.
- The identified relationships may support preliminary selection of glass color and replacement level, but they should not replace certification testing. Because only one source of each glass color, one particle-size distribution, and laboratory-scale cylindrical specimens were investigated, the proposed screening framework requires validation using independent waste glass streams, including consideration of their purity, coatings, impurities, and particle characteristics.
- The results support further development of color-sorted waste glass as a secondary raw material for autoclaved masonry products and may inform recycled glass quality criteria, pilot-scale programs, recycled content guidelines, and green public procurement. Before use in load-bearing walls can be recommended, thermal conductivity and frost resistance must be evaluated using full-size masonry units in accordance with applicable product standards.

## Figures and Tables

**Table 1:** Components of the lime–silica mixture.

Components	Origin of Material			Parameter/Information
Lime	supplied by Trzuskawica S.A. (located in Sitkówka, Poland), part of Cement Roadstone Holding (CRH) the international building materials group			CaO + MgO ≥ 91%, MgO ≤ 2%, CO_2_ ≤ 3%, SO_3_ ≤ 0.5%; Bulk density: 590 kg/m^3^
Water	potable water			conforming to PN-EN 1008:2004, the Polish identical adoption of EN 1008:2002 [20] was used
Quartz sand	sand used in the production of silicate products; deposits at the Ludynia silicate production plant (Świętokrzyskie Voivodeship, Poland)			fraction: 2.5–0.5 mm = 19%, 0.5–0.05 mm = 81% density: 2650 kg/m^3^
Waste packaging glass	colorless glass	green glass	brown glass	the preparation of the glass involved: removing the caps and labels, washing, drying, and grinding it into particle sizes characteristic of the quartz sand used, since the aggregate was replaced in a 1:1 weight ratio.
	household jars	green bottles	brown bottles	

**Table 2:** Chemical composition of aggregates used in the tests [wt.%].

Chemical Composition	Sand	Colorless Glass (WG)	Brown Glass (BG)	Green Glass (GG)
SiO_2_	97.59	72.53	71.56	72.11
Na_2_O	0.76	13.03	13.13	13.15
CaO	1.16	10.53	10.28	10.01
Al_2_O_3_	0.00	1.93	1.72	1.73
MgO	0.01	1.19	2.14	1.44
K_2_O	0.06	0.36	0.47	0.57
SO_3_	0.02	0.14	0.03	0.02
Fe_2_O_3_	0.02	0.08	0.41	0.43
TiO_2_		0.07	0.11	0.14
SrO		0.05	0.02	0.04
ZrO_2_		0.02	0.02	0.02
P_2_O_5_		0.01	0.01	0.01
Mn_2_O_3_		0.01	0.05	0.03
Cr_2_O_3_		0.00	0.04	0.25

**Table 3:** Testing methods used in the research.

Type of the Research	Testing Methods	
Compressive strength	After autoclaving, the specimens were stored under laboratory conditions for 21 days. Their compressive strength was then determined using a hydraulic testing machine equipped with a CONTROLS 50–C9030 compression device (CONTROLS S.p.A., Liscate, Milan, Italy), in accordance with PN-EN 772-1+A1:2015-10 [23]. The specimens were loaded continuously until failure at a rate corresponding to an increase in compressive stress of 0.5 MPa/s.	
Volumetric density	The calculations were performed using Equation (1):	
	$\rho =\frac{m_{d}}{V_{0}}$; [kg/m^3^]	(1)
	where $m_{d}$-dry sample mass, $V_{0}$-sample volume including the pores. The testing procedure followed the method described in [17]. An AXIS AG500C balance (AXIS, Warsaw, Poland) was used for the measurements.	
Water absorption	EN 772-21:2011 [24] was selected because the investigated lime–silica composites are laboratory-scale analogs of calcium silicate masonry units covered by this standard. The procedure was modified only with respect to the cylindrical specimen geometry; therefore, the results are comparative and do not constitute product certification. Water absorption was calculated using Equation (2):	
	w= ($m_{1}-m_{d}$)/$m_{d}$ × 100; [%]	(2)
	where $m_{d}$—mass of the sample after drying, and $m_{1}$—mass of the sample after soaking	
Open porosity	Open porosity was calculated based on the results obtained during the volumetric density test, using Equation (3):	
	$P_{o}=\frac{m_{1}-m_{d}}{m_{1}-m_{2}}$; [%]	(3)

**Table 4:** Spearman’s rank correlation coefficients r_s_ and corresponding two-sided *p*-values for the properties of lime–silica products containing colorless (WG), brown (BG), and green (GG) waste packaging glass.

Relationship	WG r_s_ (*p*)	BG r_s_ (*p*)	GG r_s_ (*p*)	n
Compressive strength–volumetric density	−0.925 (<0.001)	−0.903 (<0.001)	−0.870 (<0.001)	24
Compressive strength–water absorption	0.930 (<0.001)	0.929 (<0.001)	0.943 (<0.001)	24
Compressive strength–open porosity	0.937 (<0.001)	0.919 (<0.001)	0.897 (<0.001)	24
Volumetric density–water absorption	−0.938 (<0.001)	−0.909 (<0.001)	−0.845 (<0.001)	24
Volumetric density–open porosity	−0.905 (<0.001)	−0.901 (<0.001)	−0.780 (<0.001)	24
Water absorption–open porosity	0.950 (<0.001)	0.932 (<0.001)	0.903 (<0.001)	24

**Table 5:** Standard deviations (SDs) of the measured properties for the reference and waste glass-modified lime–silica product series (*n* = 6 individual measurements per property and series).

Sample Series	Compressive Strength	Volumetric Density	Water Absorption	Open Porosity
R	0.034	27.078	0.20	0.233
WG33	0.053	20.375	0.07	0.225
WG66	0.151	16.416	0.13	0.226
WG100	0.081	13.022	0.13	0.251
BG33	0.171	31.992	0.17	0.199
BG66	0.210	18.443	0.14	0.090
BG100	0.204	23.469	0.18	0.325
GG33	0.191	15.845	0.12	0.197
GG66	0.069	16.865	0.18	0.231
GG100	0.079	18.944	0.30	0.326

## Data Availability

The original contributions presented in this study are included in the article. Further inquiries can be directed to the corresponding author.

## Abbreviations

The following abbreviations are used in this manuscript:

AAMs	Alkali-activated materials
ASR	Alkali-silica reaction
BG	Brown waste packaging glass
C-S-H	Calcium silicate hydrate
CRH	Cement roadstone holding
EDS	Energy-dispersive X-ray spectroscopy
EN	European standard
EU	European Union
GG	Green waste packaging glass
ISO	International Organization for Standardization
PN-EN	Polish Standard adopting a European Standard
R	Reference sample series without waste glass
SD	Standard deviation
SEM	Scanning electron microscopy
WG	Colorless waste packaging glass
XRD	X-ray diffraction
